# Gastric leishmaniasis in an immunocompromised patient: a case report

**DOI:** 10.1128/asmcr.00136-26

**Published:** 2026-10-12

**Authors:** Asad Ur Rehman, Alexander Banerjee, Bennett Christie-Nguyen, Antonio Galvao Neto, Violeta Chavez, Rossana Kazemimood

**Affiliations:** 1 Department of Pathology and Laboratory Medicine, McGovern Medical School at the University of Texas Health Science Center 200590, Houston, Texas, USA; Pattern Bioscience, Austin, Texas, USA

**Keywords:** HIV, AIDS, *Leishmania *spp., gastric leishmaniasis, visceral leishmaniasis

## Abstract

**Background:**

Leishmaniasis is a tropical infection that usually presents as a cutaneous, mucocutaneous, or visceral disease. The most common organs involved in visceral leishmaniasis are the liver, spleen, and bone marrow; involvement of the stomach is rare, with only a few cases reported to date.

**Case Summary:**

We present a 45-year-old male with acquired immunodeficiency syndrome (AIDS) and a history of visceral leishmaniasis. The patient was admitted to the hospital due to pancytopenia. During the workup, low vitamin B12 levels and elevated intrinsic factor antibodies raised suspicion of atrophic gastritis, prompting endoscopy for gastric mapping.

**Conclusion:**

The patient was diagnosed with gastric leishmaniasis. Several macrophages containing intracellular amastigotes were seen. PCR analysis of the bone marrow biopsy identified the organism as *Leishmania donovani/infantum/chagasi*. This case underscores the critical value of histologic examination, with the diagnosis relying on recognition based on routine histology, confirmed by molecular testing.

## INTRODUCTION

Leishmaniasis is a parasitic disease caused by infection with obligate intracellular protozoa of the genera *Leishmania* and *Endotrypanum* (1–4). More than 20 *Leishmania* species infect humans and are transmitted through the bites of infected female phlebotomine sand flies (2). It commonly occurs in tropical and subtropical regions and presents in three main clinical forms: cutaneous, mucosal, and visceral, depending on the species and site of infection (2–4). The most severe form of the disease is visceral leishmaniasis (VL), which typically involves the reticuloendothelial system and presents with prolonged fever, hepatosplenomegaly, weight loss, and pancytopenia (2, 5). If left untreated, VL is always fatal (2, 3). Involvement of the gastrointestinal (GI) tract, particularly the stomach, is rare and usually observed in profoundly immunocompromised patients such as those with advanced human immunodeficiency virus (HIV) infection, notably with acquired immunodeficiency syndrome (AIDS) (2–4). Because endoscopic changes are often nonspecific, diagnosis relies on the microscopic identification of amastigotes within macrophages in biopsy tissue (5, 6). Recognition of this entity in gastric biopsy is crucial for pathologists, as it can represent the first evidence of disseminated disease and directly influence management and follow-up.

## CASE PRESENTATION

The patient is a 45-year-old male originally from Honduras, who has lived in the United States since 2003. He has a history of HIV infection, which was diagnosed in 2014; however, he was lost to follow-up. In 2015, he was diagnosed with advanced HIV/AIDS, with severe immunosuppression (CD4 count of 9 cells/µL with a standard range of 431–1,623 cells/µL) and an HIV viral load of 52,000 copies/mL. He was also diagnosed with syphilis at the same time and received treatment; however, the treatment course was incomplete, necessitating multiple courses of intramuscular benzathine penicillin (2.4 million units).

Antiretroviral therapy (ART) was initiated with elvitegravir/cobicistat/emtricitabine/tenofovir alafenamide. Prophylaxis against opportunistic infections with trimethoprim–sulfamethoxazole and azithromycin (1,200 mg weekly) was initiated. ART resulted in durable viral suppression, although the CD4 count remained persistently below 200 cells/µL for several years (Table 1).

**TABLE 1:** CD4 absolute count and HIV-1 RNA detection status from 2019 till last follow-up

Laboratory test	8/2019	7/2020	9/2021	8/2022	11/2023	03/2024	8/2024	11/2025	Reference range
CD4 absolute	95 cells/µL	86 cells/µL	146 cells/µL	117 cells/µL	73 cells/µL	70 cells/µL	200 cells/µL	119 cells/µL	431–1,623 cells/µL
HIV-1 RNA detection	Not detected	Not detected	Not detected	Not detected	Not detected	Not detected	Not detected	Not detected	Not detected

Late in 2016, he developed oropharyngeal candidiasis, onychomycosis, and a swollen upper lip. Biopsy of the left maxillary oral mucosa revealed oral histoplasmosis. He was treated with itraconazole, completing 1 year of therapy, and subsequently maintained on suppressive therapy with itraconazole 200 mg daily. The ART treatment transitioned to abacavir/dolutegravir/lamivudine due to drug interactions when antifungal therapy started.

In 2017, the patient developed hepatosplenomegaly and lymphadenopathy, which were considered reactive, along with a chronic generalized maculopapular rash that was clinically suspected to represent secondary syphilis. In 2018, a skin biopsy was performed, and the results showed a diffuse dermal granulomatous infiltrate with parasitized macrophages exhibiting morphological features characteristic of *Leishmania* spp. Following confirmation of visceral leishmaniasis, the patient was hospitalized and treated with five consecutive doses of liposomal amphotericin B (300 mg per dose), followed by monthly maintenance infusions for suppressive therapy. However, interval imaging revealed persistent lymphadenopathy and splenomegaly.

**Fig 1:**
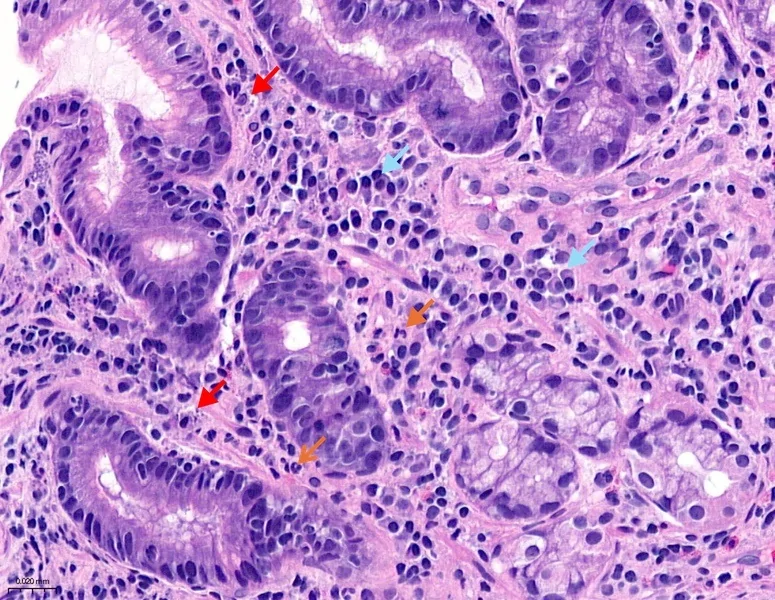
Active chronic gastritis, including neutrophils (orange arrows), plasma cells (blue arrows), and macrophages (red arrows) within the lamina propria. H&E, 400× magnification.

**Fig 2:**
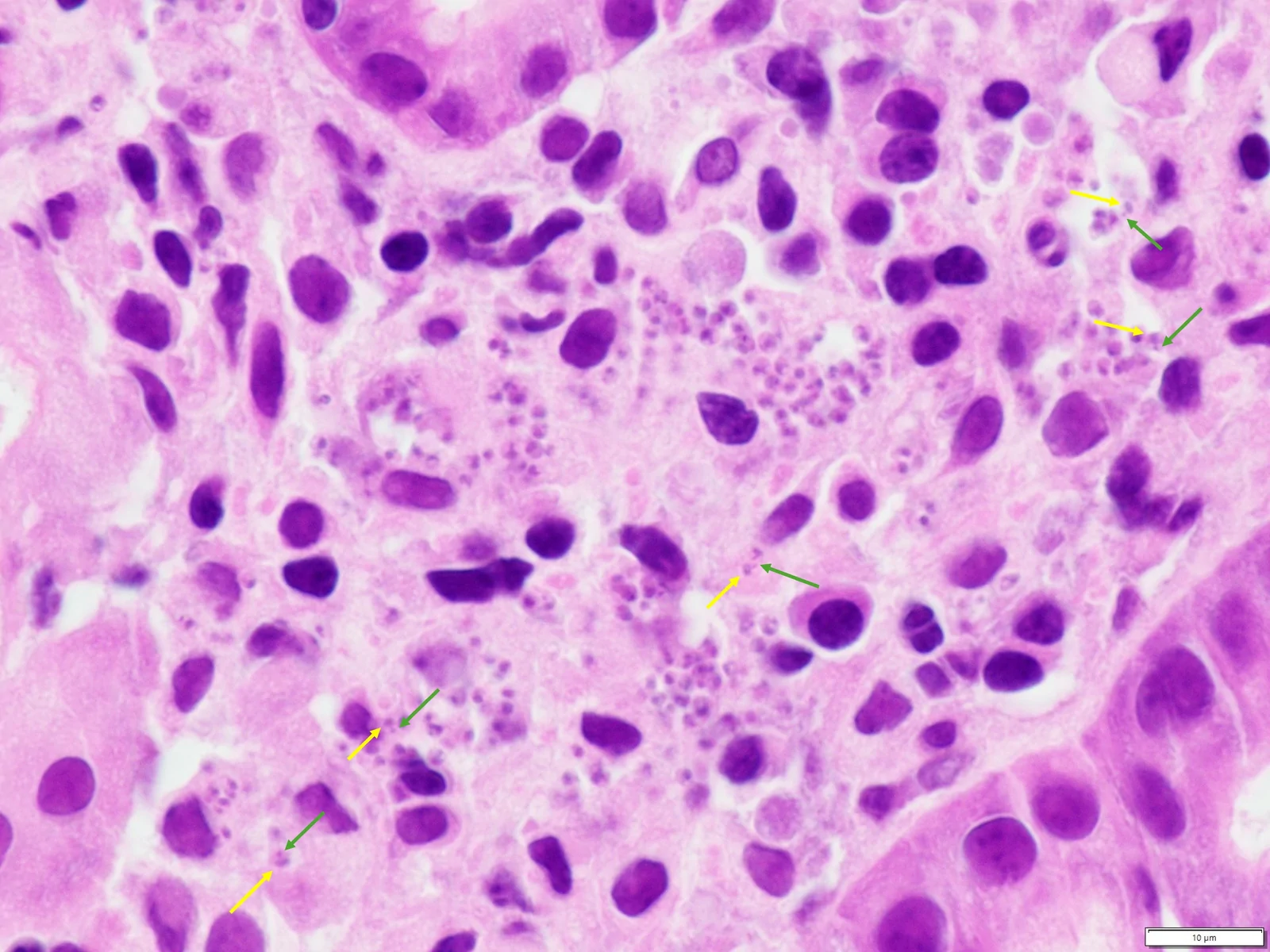
Macrophages containing numerous *Leishmania* amastigotes that are spherical to ovoid and measure 1–5 µm long by 1–2 µm, with a nucleus (yellow arrow) and a kinetoplast that is rod/disc shaped (green arrow). Taken in 2024. H&E staining, 500× magnification (oil immersion).

**Fig 3:**
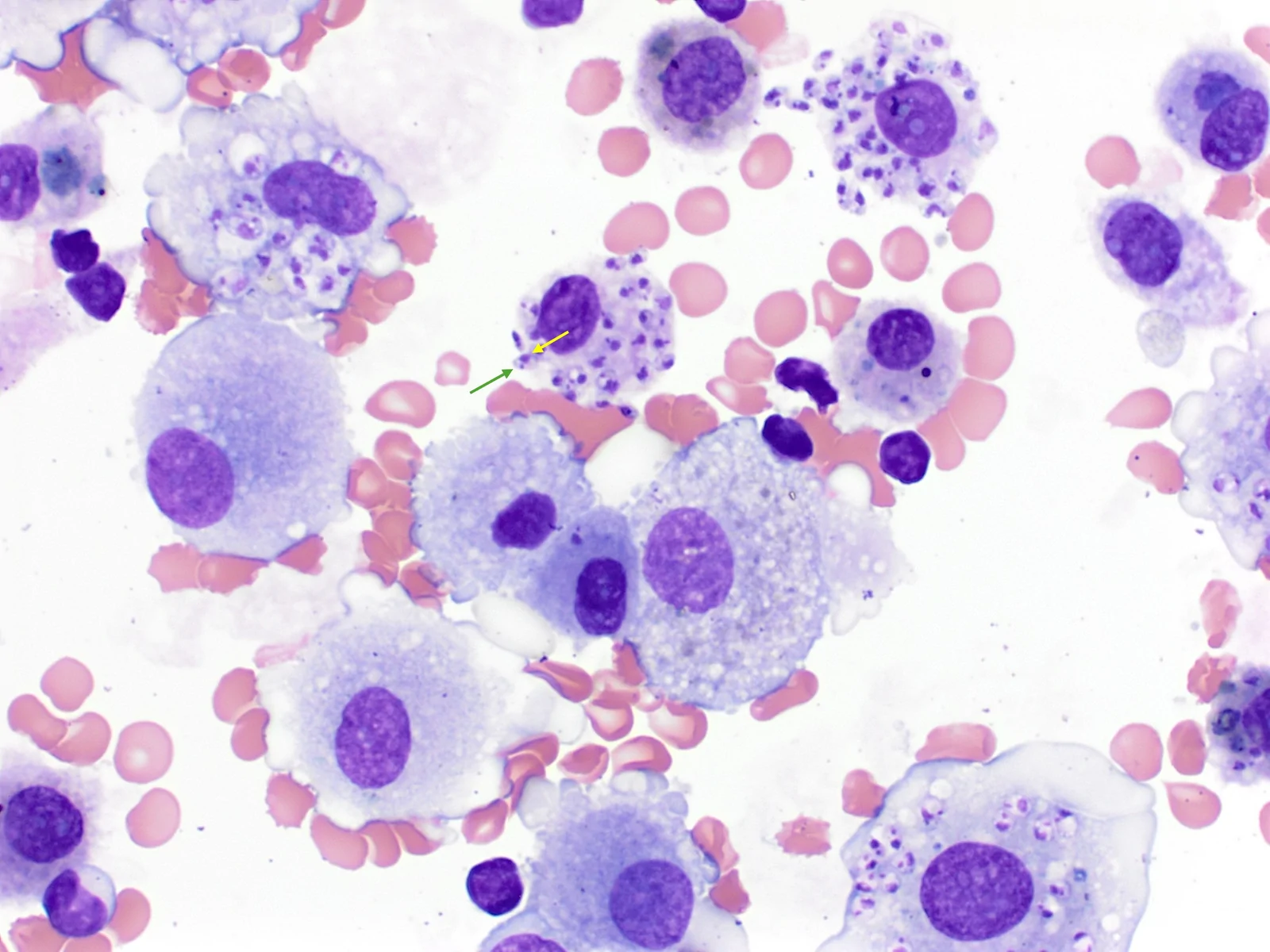
Amastigotes within macrophages (arrows) on Giemsa stain from a BAL (bronchoalveolar lavage), with a nucleus (yellow arrow) and a kinetoplast that are rod/disc shaped (green arrow). Taken in 2024. 400× magnification.

The patient was admitted to the hospital in early 2024 due to pancytopenia (Table 2). A computed tomography (CT) scan of the abdomen demonstrated marked splenomegaly, mild to moderate hepatomegaly, and mesenteric lymphadenopathy. During the workup, low vitamin B12 levels (133 pg/mL with a standard range of 180–914 pg/mL) and elevated intrinsic factor antibodies (9.5 AU/mL with a standard range of 0.0–1.1 AU/mL) raised the possibility of atrophic gastritis, prompting endoscopy for gastric mapping and biopsies. Endoscopic evaluation revealed mild mucosal abnormalities in the gastric body and antrum, with an irregular Z-line. Gastric biopsies from the body and antrum demonstrated acute inflammation with neutrophils and chronic inflammatory infiltrates with plasma cells and macrophages (Fig. 1). Many macrophages in the lamina propria contained clustered amastigotes with large nuclei and kinetoplasts, consistent with *Leishmania* organisms (Fig. 2). *Helicobacter pylori* was confirmed by immunohistochemical staining. Histochemical (H&E, Giemsa) and immunohistochemical stains were performed using standard clinical laboratory protocols. Polymerase chain reaction (PCR) analysis of the bone marrow by the Centers for Disease Control and Prevention (CDC), followed by sequencing, identified the organism as *Leishmania donovani/infantum/chagasi*. In addition, the patient underwent bronchoalveolar lavage, and *Leishmania* was identified with distinct nuclei and kinetoplasts on Giemsa stain (Fig. 3). This admission confirmed gastric involvement in relapsing visceral leishmaniasis.

**TABLE 2:** Complete blood count at the time of admission

Laboratory test	Result at time of admission	Reference range
WBC	4.1 × 10³/µL	4.5 × 10³–12 × 10³/µL
HGB	4.9 g/dL	14–18 g/dL
Platelets	84 × 10³/µL	150 × 10³–400 × 10³/µL

The patient’s symptoms were consistent with those of histoplasmosis. *Histoplasma* can also be observed in macrophages and can resemble amastigotes. However, *Leishmania* spp. have a distinct kinetoplast, which is not present in *Histoplasma*. Grocott’s methenamine silver stain, which would appear positive with the presence of *Histoplasma*, was negative, also weighed against the diagnosis of histoplasmosis. Additionally, urine antigen testing for *Histoplasma* was negative.

An alternative diagnosis for gastric and bone marrow amastigotes is *Trypanosoma cruzi*, particularly given the patient’s Latin American origin and immunocompromised status. However, several clinical and pathological features in this case were incompatible with chronic Chagas disease. In chronic Chagas disease, the most commonly affected organ is the heart, leading to dilated cardiomyopathy, arrhythmia, and thromboembolic complications (7). Gastrointestinal manifestations of chronic Chagas disease include megaesophagus and megacolon (8). In addition, *T. cruzi* affects cardiac and gastrointestinal smooth muscles and enteric neurons, whereas *Leishmania* species cause visceral leishmaniasis, which involves the reticuloendothelial system, including macrophages (8). Amastigotes of *Leishmania* spp. and *T. cruzi* are indistinguishable, as they both have a nucleus and kinetoplast. However, the clinical and histological findings in our case were not compatible with chronic Chagas disease. Serological testing for *Trypanosoma cruzi* IgG was negative.

The patient was treated for *H. pylori* infection, and amphotericin B and miltefosine were continued for relapsing VL. The last recorded visit was in late 2025. The patient was asymptomatic with undetectable HIV viral loads, and the CD4 count remained persistently low (119 cells/µL; reference range 431–1,623 cells/µL) despite ART adherence. The patient is still receiving monthly amphotericin B infusions for VL-suppressive therapy. Table 3 summarizes the timeline of events.

**TABLE 3:** Timeline of key clinical events

Date	Key clinical event
2003	Immigrated to the United States from Honduras
2014	Diagnosed with HIV infection and subsequently lost to follow-up after diagnosis
2015	The patient was diagnosed with advanced HIV/AIDS (CD4 9 cells/µL; HIV viral load 52,000 copies/mL) and syphilis; antiretroviral therapy and opportunistic infection prophylaxis were initiated
2016	The patient developed oropharyngeal candidiasis, onychomycosis, and oral histoplasmosis (maxillary mucosal biopsy) and was treated with itraconazole
2017	The patient developed hepatosplenomegaly, lymphadenopathy, and generalized maculopapular rash
2018	Skin biopsy showed parasitized macrophages consistent with *Leishmania* spp.; visceral leishmaniasis was confirmed and treated with liposomal amphotericin B, followed by maintenance infusions
2024	Admitted with pancytopenia, low vitamin B12 levels, and elevated intrinsic factor antibodies underwent endoscopy, revealing gastric leishmaniasis with *Helicobacter pylori* coinfection; bone marrow PCR confirmed *Leishmania donovani/infantum/chagasi*, and BAL was positive for *Leishmania* spp.
2025	At the last follow-up, the patient was asymptomatic, had an undetectable HIV viral load and a persistently low CD4 count (119 cells/µL), and continued receiving monthly amphotericin B suppressive therapy for VL

## DISCUSSION

VL remains endemic in many regions, including South Asia, East Africa, and the Mediterranean (2–4). In the Americas, VL occurs primarily in Brazil, with sporadic cases elsewhere in Central and South America and rare autochthonous transmission in the United States, particularly in Texas (2–4).

Diagnosis of visceral leishmaniasis (VL) requires the demonstration of the parasite and often relies on a combination of histopathologic and laboratory methods, especially in immunocompromised patients. Histologic examination can play an important role in diagnosis because intracellular amastigotes within macrophages can be observed in tissue biopsies. Usually, the bone marrow is the site of biopsy for VL, but any organ involved by VL can be biopsied, including the liver, lymph nodes, or GI tract. Immunohistochemistry for *Leishmania* can help detect organisms, but is not commercially available in areas where *Leishmania* is not endemic, such as the United States (9–12). Molecular testing has high sensitivity and specificity for diagnosis, but not all reference laboratories can differentiate species, which is useful for treatment.

Other tests are available for *Leishmania* spp.; however, they have lower sensitivity and specificity. Serological tests for *Leishmania* can be performed using enzyme immunoassay. *Leishmania* antibody (IgG) can support the diagnosis of VL. However, some patients with cutaneous leishmaniasis may have undetectable IgG levels, and there is cross-reactivity with those with Chagas disease (13, 14). In addition, the detection of IgG antibodies cannot distinguish between current and past infections (14, 15). Culture is rarely used because of its low sensitivity and prolonged turnaround time (12, 16).

Immunocompromised individuals, particularly those with advanced HIV/AIDS, are at increased risk of atypical VL presentations (2–4, 17–19). Gastric involvement has been reported in this population (5, 20–23). Most reported cases of gastric leishmaniasis occur as part of a disseminated disease rather than as an isolated infection (5, 20, 22). A review of 15 cases of HIV-associated gastrointestinal leishmaniasis (5 original and 10 previously reported) found that nearly all patients had CD4 counts below 200 cells/µL; most had digestive symptoms such as diarrhea, dysphagia, or abdominal pain; the duodenum (90%) and stomach (75%) were the most frequently involved sites; and endoscopy was macroscopically normal in nearly half of the cases (21). Subsequent single-case reports illustrate a similarly variable presentation, ranging from an incidental, asymptomatic finding on routine histology (5) to relapsing gastric disease despite amphotericin B and antiretroviral therapy (20) to a fatal outcome (23), underscoring the diagnostic subtlety of this entity and the importance of histologic vigilance in immunocompromised hosts. Profound immunosuppression, particularly in advanced HIV/AIDS, predisposes patients to disseminated and relapsing VL (5, 20–22). CD4 depletion impairs cellular immunity, allowing *Leishmania* to persist despite therapy (21). Standard treatment with amphotericin B and maintenance therapy is essential, although relapse remains common in severely immunocompromised hosts, whereas sustained CD4 recovery has been associated with more durable remission (20, 21, 24).

*Leishmania* parasites typically infect macrophages within the reticuloendothelial system and produce classic VL features (20–22). In patients with severe immunosuppression, impaired T cell-mediated immunity facilitates dissemination to uncommon sites, including the gastrointestinal tract. Gastric involvement may occur without systemic symptoms, making biopsy essential for the diagnosis. Our patient, a male with advanced HIV infection and low CD4 counts, exemplifies this high-risk population.

Clinical manifestations are often nonspecific, ranging from dyspepsia to epigastric discomfort, and systemic VL features may be absent (5, 20, 22). Endoscopic findings are subtle and nonspecific, mimicking chronic gastritis, lymphoma, or infections such as cytomegalovirus and *H. pylori* (5, 20–22). In this case, gastric biopsies revealed macrophage-rich infiltrates containing *Leishmania* amastigotes, which were confirmed using Giemsa staining. Bone marrow was sent to the Centers for Disease Control and Prevention (CDC), where PCR and DNA sequencing were performed, resulting in a diagnosis of *Leishmania donovani/infantum/chagasi*. Coinfection with *H. pylori*, as seen in this patient, may exacerbate mucosal inflammation and complicate the interpretation.

Gastric leishmaniasis is a rare and under-recognized manifestation of VL in immunocompromised patients. Visceral leishmaniasis is diagnosed by detecting *Leishmania* parasites or molecular testing of the bone marrow. PCR was positive in the bone marrow, confirming VL, and histological examination provided evidence of gastric leishmaniasis (5, 22). These tools are critical for timely diagnosis, appropriate therapy, and relapse monitoring in high-risk populations.

### Conclusion

Gastric leishmaniasis is a rare manifestation of visceral disease that occurs in patients with severe immunodeficiency. Recognition relies on careful histopathological evaluation, as endoscopic findings are nonspecific, and concurrent infections, such as *H. pylori*, may obscure the diagnosis. Identifying amastigotes in gastric biopsies should prompt consideration of disseminated disease and further systemic evaluation. This case emphasizes the critical role of pathologists in detecting unusual infectious agents in routine hematoxylin and eosin (H&E) sections, underscoring the importance of vigilance and awareness of potential diagnostic pitfalls in immunocompromised hosts.

## Data Availability

The scanned de-identified slides are accessible upon request.
